# Abundance of Aβ_5-x_ like immunoreactivity in transgenic 5XFAD, APP/PS1KI and 3xTG mice, sporadic and familial Alzheimer’s disease

**DOI:** 10.1186/1750-1326-9-13

**Published:** 2014-04-02

**Authors:** Erika Avendaño Guzmán, Yvonne Bouter, Bernhard C Richard, Lars Lannfelt, Martin Ingelsson, Anders Paetau, Auli Verkkoniemi-Ahola, Oliver Wirths, Thomas A Bayer

**Affiliations:** 1 Division of Molecular Psychiatry Department of Psychiatry, University Medicine Goettingen, D-37075, von-Siebold-Str. 5, Goettingen 37075, Germany; 2 Uppsala University, Department of Public Health/Geriatrics, Uppsala, Sweden; 3 Department of Pathology, University and University Hospital of Helsinki, Helsinki, Finland; 4 Department of Neurology, Helsinki University Central Hospital, Helsinki, Finland

**Keywords:** Aβ_5-x_, Amyloid plaques, 5XFAD, 3xTG, APP/PS1KI, Vascular deposits, N-truncated Aβ

## Abstract

**Background:**

According to the modified amyloid hypothesis the main event in the pathogenesis of Alzheimer’s disease (AD) is the deposition of neurotoxic amyloid β-peptide (Aβ) within neurons. Additionally to full-length peptides, a great diversity of N-truncated Aβ variants is derived from the larger amyloid precursor protein (APP). Vast evidence suggests that Aβ_x-42_ isoforms play an important role triggering neurodegeneration due to its high abundance, amyloidogenic propensity and toxicity. Although N-truncated and Aβ_x-42_ species have been pointed as crucial players in AD etiology, the Aβ_5-x_ isoforms have not received much attention.

**Results:**

The present study is the first to show immunohistochemical evidence of Aβ_5-x_ in familial cases of AD (FAD) and its distribution in APP/PS1KI, 5XFAD and 3xTG transgenic mouse models. In order to probe Aβ_5-x_ peptides we generated the AB5-3 antibody. Positive plaques and congophilic amyloid angiopathy (CAA) were observed among all the FAD cases tested carrying either APP or presenilin 1 (PS1) mutations and most of the sporadic cases of AD (SAD). Different patterns of Aβ_5-x_ distribution were found in the mouse models carrying different combinations of autosomal mutations in the APP, PS1 and Tau genes. All of them showed extracellular Aβ deposits but none CAA. Additionally, they were all affected by a severe amyloid pathology in the hippocampus among other areas. Interestingly, neither 5XFAD nor APP/PS1KI showed any evidence for intraneuronal Aβ_5-x_.

**Conclusions:**

Different degrees of Aβ_5-x_ accumulations can be found in the transgenic AD mouse models and human cases expressing the sporadic or the familial form of the disease. Due to the lack of intracellular Aβ_5-x,_ these isoforms might not be contributing to early mechanisms in the cascade of events triggering AD pathology. Brain sections obtained from SAD cases showed higher Aβ_5-x_–immunoreactivity in vascular deposits than in extracellular plaques, while both are equally important in the FAD cases. The difference may rely on alternative mechanisms involving Aβ_5-x_ peptides and operating in a divergent way in the late and early onset forms of the disease.

## Background

Alzheimer’s disease (AD) is the most common type of dementia worldwide. It is characterized by the accumulation of specific proteins, namely tau and amyloid-beta protein (Aβ). In fact, these proteins are essential to confirm an AD diagnosis, given that the two major histopathological hallmarks are extracellular amyloid-β plaques surrounded by dystrophic neurites and intracellular neurofibrillary tangles. Furthermore, AD is characterized by neuronal loss, gliosis and congophilic angiopathy mainly affecting the cortex and the hippocampal formation [1].

The amyloid hypothesis considers the accumulation of Aβ peptides as the central and triggering event in AD [2,3]. The formation of neurotoxic oligomers and larger assemblies of Aβ are thought to be the product of an imbalance in its production and clearance [4]. Aβ derives from the larger amyloid precursor protein (APP) by proteolytic cleavage of different secretase enzymes. The combined activity of β- and γ-secretase activities releases Aβ peptides of various lengths [5]. The γ-secretase comprises a high molecular weight complex that depends on presenilin-1 and -2 (PS1, PS2) activity to cleave within the transmembrane domain of APP to generate Aβ peptides and is composed of four integral membrane proteins: presenilin, nicastrin, Aph-1 and Pen-2 [6]. Supporting the amyloid hypothesis, autosomal dominant mutations in APP, PS1 and PS2 genes cause familial early onset AD mainly by increasing the production of Aβ_x-42_[7]. However, advancing in age is considered the most prevalent risk factor for Aβ accumulation and most of the cases have a late onset. These cases are classified as sporadic AD.

Extracellular plaques are formed by Aβ peptides with different C–termini ranging from position 38 to 43 [5]. Since the 42 amino acid isoforms Aβ_x-42_ are highly susceptible to aggregate and to form oligomer and amyloid fibrils [8], it is considered the main plaque component and the initiator of plaque formation in AD pathogenesis [9]. In addition to the most prevalent species Aβ_x-40_ and Aβ_x-42_, other isoforms such as Aβ_x-38_ has been reported in different mouse models, FAD cases due to mutations in APP and PS1 and in the vascular Aβ deposits of SAD cases [10,11]. Aβ species ending at position 43 have shown to be potently amyloidogenic and abundant [12]. Additionally to the full length Aβ peptides starting with N-terminal aspartate at position 1, different N-truncated isoforms have been demonstrated to be as abundant as toxic due to their capacity to rapidly form stable aggregates [13]. Not much is known about the N-terminally truncated Aβ_5-x_ present in the amyloid pathology of AD brains. It has been suggested that these Aβ variants are preferentially formed by an alternative cleavage of APP involving caspase activity [14].

The aim of the present work was to characterize our recently generated antibody as a tool to study the presence of Aβ_5-x_ in different AD transgenic mouse models and human cases, including sporadic AD and familial AD cases carrying either APP or PS1 mutations.

## Material and methods

### Antibodies

The rabbit polyclonal antibodies AB5-3 and 24311 were generated by PSL Heidelberg (will be distributed by T.A.B. upon request). 24311 recognizes pan-Aβ (epitope Aβ_4–40_) and AB5-3 recognizes the free N-terminus of Aβ_5–42_ (epitope Aβ_5–42_). The polyclonal rabbit antibody Aβ_42_ was purchase from Synaptic Systems, Goettingen. The monoclonal antibody 4G8 is a pan-Aβ antibody (epitope 17–24; Covance). All antibodies were affinity purified.

### Human brain samples

We examined the brains of 12 sporadic AD and 9 non-demented control patients (Table 1). In addition, we studied brains from familial AD patients either carrying APP or PS1 mutations. The APP mutations KM670/671NL (Swedish) [15] and E693G (Arctic) [16,17] were included as well as three different subjects carrying the PS1 delta exon 9 mutation [18]. Human brain samples were obtained from the Netherlands Brain Bank (NBB), Hopital de la Salpetriere (a generous gift of C. Duyckaerts and V. Sazdovitch), University Hospital Helsinki and from Uppsala University. Definite diagnosis was based on established criteria and written informed consent was obtained from all subjects.

**Table 1:** Demographic data and semiquantitative analysis of antibody stainings in SAD, FAD and non-demented control cases

**Case**	**Age**	**Gender**	**Braak stage**	**Aβ**_**5-x**_		**Aβ**	
				**SP**	**CAA**	**SP**	**CAA**
**Sporadic AD cases:**							
AD-1	88	F	IV	+	-	+++	++
AD-2	84	F	IV	+	-	+++	-
AD-3	86	M	IV	+	++	++	+++
AD-4	88	F	IV	+	-	++	-
AD-5	84	F	IV	+	++	+++	++
AD-6	93	M	IV	-	+	++	+++
AD-7	92	M	IV	-	+	+++	++
AD-8	92	F	IV	+	++	+++	+++
AD-9	91	F	IV	+	++	+++	+++
AD-10	91	M	IV	+	+	++	+++
AD-11	92	F	IV	-	+	++	+++
AD-12	91	F	IV	-	-	+++	++
**Non-demented control cases:**							
NDC-1	70	M	0	-	-	-	-
NDC-2	90	F	I	+	+	++	++
NDC-3	88	F	I	+	-	++	-
NDC-4	73	M	0	-	-	-	-
NDC-5	91	M	I	-	-	-	-
NDC-6	78	F	I	+	-	++	-
NDC-7	84	M	I	-	-	-	-
NDC-8	78	M	I	+	-	++	++
NDC-9	82	F	I	-	-	-	-
**Familial AD cases**							
APPE693G	64	M	n.a.	++	++	+++	+++
APPKM670/671NL	61	F	n.a.	++	++	+++	+++
PS1∆Ex9	69	M	n.a.	++	+	+++	+
PS1∆Ex9	61	M	n.a.	++	++	+++	+
PS1∆Ex9	64	M	n.a.	+	+	+++	+

*Abbreviations:SP* senile plaques, *CAA* congophilic amyloid angiopathy.

### Transgenic mouse brain samples

Brain sections were obtained from 3 established AD transgenic mouse models including 3xTg [19], APP/PS1KI [20] (generous gift by Dr. Laurent Pradier, Sanofi) and 5XFAD [21]. The 5XFAD mice have been crossed on C57Bl6 background [22].

### Sample preparation

The amyloid β (Aβ) variants Aβ_1–40_, Aβ_1–42_, Aβ_4–38_, Aβ_4–40_, Aβ_4–40_, Aβ_5–42_, Aβ_pE3–40_, and Aβ_pE3–42_ were purchased from Peptide Specialty Laboratory (PSL, Heidelberg) and used without further purification as previously described [23]. Briefly, Aβ peptides were dissolved in 10 mM NaOH at a concentration of 1 mg/mL and aliquoted in 50 μl volumes. After flash freezing in liquid nitrogen, the aliquots were stored at -80°C until use.

### Analysis of AB5-3 using dot blotting

2 μl of different Aβ isoforms were spotted on a nitrocellulose membrane in different quantities (2, 1, 0.5, 0.25 and 0.125 μg). The membrane was dried for 20 minutes and blocked with 10% non-fat dry milk in TBS-T (10 mM Tris pH 8.0, 150 mM NaCl, 0.05% Tween 20) for 30 minutes. Primary antibodies AB5-3 (diluted 1:500) and 24311 (pan-Aβ, 1:500 were incubated overnight at 4°C. Running and transfer buffers were applied according to the manufacturer. The blots were developed using enhanced chemiluminescence according to the manufacturer (Roth). Horse radish conjugated swine anti-rabbit antibody was used as a secondary antibody (1:3,000, Dianova).

### Electrophoresis and Western blotting of synthetic peptides

For Western blot analysis under reducing conditions, 2 μg of each Aβ peptide was loaded per lane after heating at 95°C for 5 minutes on 4 - 12% Tris-Tricin VarioGels (Anamed), transferred to 0.45 μm nitrocellulose membranes (GE Healthcare) and detected using the primary antibodies AB5-3 (diluted 1:500) and 4G8 (diluted 1:2000). Running and transfer buffers were applied according to the manufacturer. The blots were developed using enhanced chemiluminescence according to the manufacturer (Roth).

### Immunohistochemistry

Immunohistochemistry was performed on 4 μm sagittal paraffin sections, as described previously [23]. In brief, sections were deparaffinized in xylene, followed by rehydration in a series of ethanol (70%, 95%, and 100%). After treatment with 0.3% H_2_O_2_ in 0.01 M PBS to block endogenous peroxidases, sections were boiled in 0.01 M citrate buffer pH 6.0 for antigen retrieval, followed by 3 min incubation in 88% formic acid. Non-specific binding sites were blocked by treatment with skim milk and fetal calf serum in PBS for 1 h at room temperature (RT), prior to the addition of the primary antibodies. Rabbit polyclonal antibodies against Aβ_5-x_ (1:100, AB5-3) and Aβ_42_ (1:500, Synaptic Systems, Goettingen, Germany), as well as mouse monoclonal 4G8 (1:5000, Covance, Dedham, MA, USA) and the rabbit polyclonal 24311 (1:500, [23]) pan-Aβ antibodies were incubated overnight in a humid chamber at RT. This was followed by incubation with the corresponding biotinylated secondary antibodies (1:200, DAKO, Glostrup, Denmark) at 37°C before visualization of the staining using the ABC method with a Vectastain kit (Vector Laboratories, Burlingame, USA) and diaminobenzidine (DAB) as chromogen. Counterstaining was carried out with hematoxylin.

## Results

### Antibody specificity

**Figure 1:**
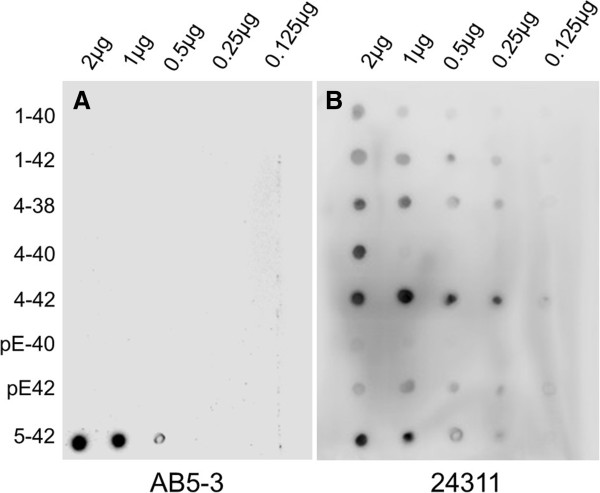
**Analysis of the AB5-3 using dot blotting.** Full length and N-truncated amyloid-β peptides were probed and spotted into a nitrocellulose membrane. **(A)** After incubation with the primary antibody AB5-3 only the Aβ_5–42_ variant was recognized indicating specificity for this peptide. **(B)** The pan-Aβ antibody 24311 recognized all Aβ variants demonstrating that all peptides were available.

**Figure 2:**
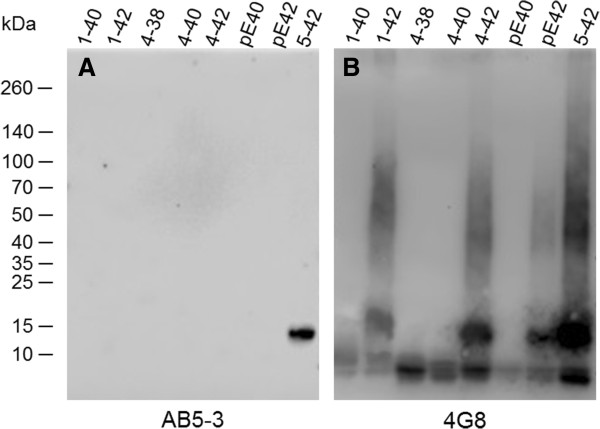
**SDS-PAGE Western blot analysis of the AB5-3 and pan-Aβ 4G8 antibodies.** Freshly dissolved synthetic Aβ variants (2 μg each) were probed to a membrane. **(A)** The polyclonal AB5-3 antibody detects Aβ5–42 trimers and tetramers while **(B)** the monoclonal pan-Aβ antibody 4G8 detects different sizes of all Aβ species.

The specificity of the polyclonal rabbit antibody AB5-3 was tested by dot blot (Figure 1) and western blot analysis (Figure 2). AB5-3 showed no cross-reactivity with full-length peptides starting with aspartate at position 1 and other N-truncated Aβ isoforms starting with pyroglutamate at position 3 or phenylalanine at position 4. Under denaturing conditions AB5-3 reacted only with Aβ_5–42,_ thus detecting ragged peptides starting with arginine at position 5. Data obtained by western blot indicates that AB5-3 is binding to low molecular weight forms of the peptide detecting only soluble Aβ_5–42_ trimers and tetramers (Figure 2).

### Aβ_5-x_ in sporadic Alzheimer’s disease

**Figure 3:**
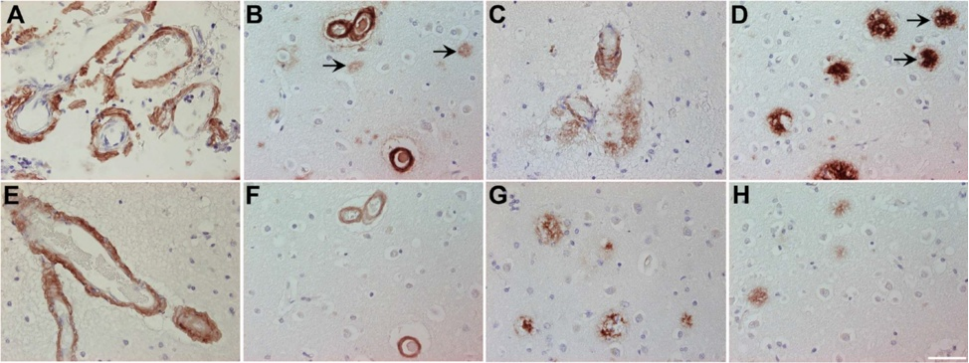
**Immunohistochemical staining pattern of Aβ**_**5-X **_**in cortex of sporadic AD human brain.** Vascular Aβ_5-x_ immunoreactivity **(A, E, F)** was detected in the majority of the SAD cases analyzed in hippocampus **(A)** and cerebral cortex **(E)**, whereas extracellular Aβ_5-x_-positive deposits **(C, G, H)** were less abundant in both brain areas **(C, D)**. The pan-Aβ antibody 24311 **(B, D)** demonstrates cerebral amyloid angiopathy (CAA) and plaque load (arrows). The same cortical region stained in a parallel section with the AB5-3 antibody **(F, H)** binds preferentially to blood vessels presenting CAA and barely recognizes plaques. Scale bar: 50 μm.

Vascular Aβ deposits of variable degree (Figure 3A, B, E, and F) were detected in two- thirds (8 of 12) of the SAD cases, as well as in one non-demented control case, using immunohistochemical analysis with the AB5-3 antibody. The extent of vascular immunoreactivity corresponded to the severity of CAA, which was assessed using the pan-Aβ antibody 4G8 (Table 1). Extracellular Aβ_5-x_-immunoreactivity was observed in 8 of 12 SAD cases (Figure 3C, D, G, and H) and in 4 control cases. Although plaque pathology and vascular deposits were present in equal number of SAD cases, vascular immunoreactivity appeared stronger than the parenchymal staining, probably due to a higher abundance of Aβ_5-x_ in vessels (Figure 3A, E and F). Using the 4G8 pan-Aβ antibody, all subjects were proven to harbor a considerable extracellular plaque load with most of them (10 of 12) showing CAA in addition (Table 1). Consequently, extracellular Aβ_5-x_ isoforms were detected in 66% of the subjects affected with plaque pathology and vascular Aβ_5-x_ immunoreactivity in 83% of the subjects affected with CAA. Both diffuse and dense core plaques were positive.

### Aβ_5-x_ in familial AD

**Figure 4:**
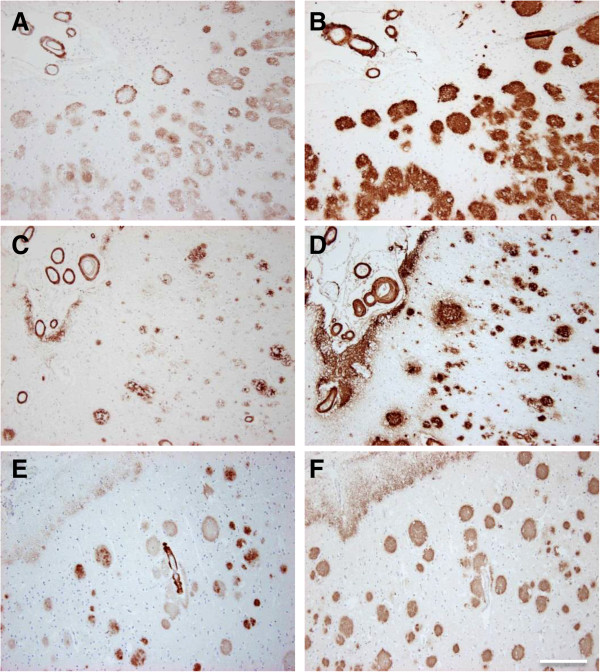
**Immunohistochemical staining pattern of Aβ**_**5-x **_**in cortex of familial AD human brain.** Vascular and parenchymal Aβ_5-x_-positive deposits were detected in parallel sections obtained from FAD cases including subjects with the Arctic **(A)** and Swedish mutations **(C)** in contrast to abundant 4G8-immuoreactivity in both cases **(B, D)**. Sections of a PS1∆E9 mutation carrier probed with the AB5-3 antibody **(E)** showed equal or higher vascular Aβ_5-x_-immunoreactivity compared to Aβ42 **(F)** whereas the Aβ_5-x_-parenchymal deposits were detected with lower intensity. Scale bar: 200 μm.

Samples from five different subjects carrying different autosomal dominant mutations, either in the APP or PS1 gene, were probed with the AB5-3 antibody. All familial cases analyzed in the present study showed prominent vascular and extracellular Aβ_5-x_-immunoreactivity. Two cases harbored APP pathogenic mutations, either located at codon 693 within the Aβ region of APP (E693G APP) known as the Arctic mutation or a double mutation at codons 670 and 671 close to the β-secretase cleavage site (KM670/671NL APP) known as the Swedish mutation. On one hand, the case carrying the Arctic mutation showed strong vascular Aβ_5-x_-immunoreactivity and abundant extracellular plaque deposits. On the other hand, brain sections obtained from the subject carrying the Swedish mutation presented a slightly stronger Aβ_5-x-_signal in the vasculature and less Aβ_5-x_-immunoreactivity in plaques (Figure 4). Both cases were compared to adjacent sections stained with the 4G8 pan-Aβ antibody which demonstrated abundant extracellular plaque pathology and CAA.

The cases harboring the PS1 mutation with the exon 9 deletion also showed abundant vascular staining and less pronounced in plaques.

Both diffuse and dense core plaques were positive.

### Aβ_5-x_ in mouse models of AD

**Figure 5:**
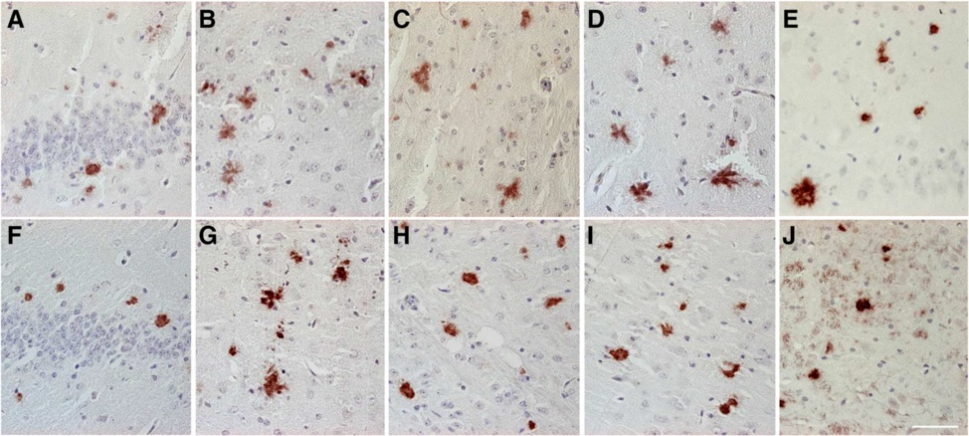
**Immunohistochemical staining pattern of Aβ**_**5-x **_**in aged APP/PS1KI, 5XFAD and 3xTg transgenic mice.** Parenchymal Aβ_5-x_ extracellular deposits were detected with the AB5-3 polyclonal antibody in the APP/PS1K1 **(A-D)**, 5XFAD **(F-I)** and 3xTg on hippocampus **(E)** and medulla **(J)**. Larger regions were affected with plaques on hippocampus **(A, F)**, cortex **(B, G)**, thalamus **(C, H)** and piriform cortex **(D, I)** in the first two mouse models. Scale bar: 50 μm.

**Figure 6:**
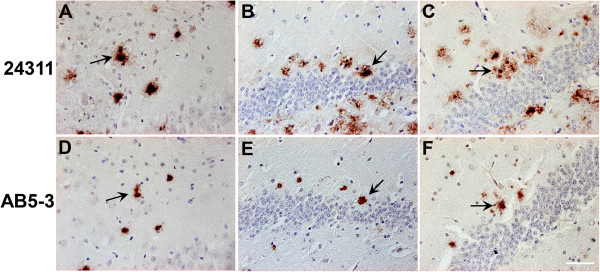
**Analysis of consecutive sections of AD mouse models using the AB5-3 and 24311 antibodies.** Extracellular plaques were detected in hippocampal brain sections of the 3xTg **(A, D)**, 5XFAD **(B, E)** and the APP/PS1K1 **(C, F)** mouse models with the AB5-3 polyclonal antibody and the 24311 pan-Aβ antibody. Parallel sections reveal Aβ_5-x_ immunoreactivity localized in the core of the plaques (arrows point to the same amyloid plaque detected by the two different antibodies). Scale bar: 50 μm.

The present report provides the first immunohistochemical analysis of Aβ_5-x_ in transgenic mouse models of AD. We analyzed three different transgenic lines, namely APP/PS1KI, 5XFAD and 3xTg (Table 2), which express different combinations of human mutant APP, PS1 and Tau. All of the mouse models showed Aβ_5-x_–positive amyloid plaques to a different extent but none of them revealed Aβ_5-x_–positive vascular deposits as expected (Figures 5 and 6). Brain sections of the 3xTg mice (18-month-old) showed amyloid deposits localized only in the subiculum of the hippocampus and the medulla (Figure 5E and J). Moreover, abundant Aβ_5-X_ positive plaques were observed in cortex, hippocampus, thalamus and piriform cortex in 10-month-old APP/PS1KI and 12-month-old 5XFAD mice (Figure 5B, E, C and F). Interestingly, the Aβ_5-x_ peptides seemed to be not very abundant and were mostly restricted to the core of plaques (Figure 6).

**Table 2:** **Transgenic AD-like mouse models in which Aβ**_**5-x**_**deposition has been analyzed**

**Mouse model**	**AβPP**	**PS1**	**Tau**	**Promoter**	**Age**	**Aβ**_**5-x**_**plaques**	**Ref.**	**Affected regions**
APP/PS1KI	Swe, Lon	M233T, L235P	-	Thy1 (APP) PS1 knock-in	10 m	++	[20]	C, Hip, Th, PiC
5XFAD	Swe, Flo, Lon	M146L, L286V	-	Thy1 (APP, PS1)	12 m	++	[21]	C, Hip, Th, PiC
3xTg	Swe	M146V	P301L	Thy1 (APP, Tau) PS1 knock-in	18 m	+	[19]	Hip

*Abbreviations:Swe* Swedish, *Flo* Florida, *Lon* London, *m* age in months, *C* Cortex, *Hip* hippocampus, *Th* thalamus, *PiC* piriform cortex.

**Figure 7:**
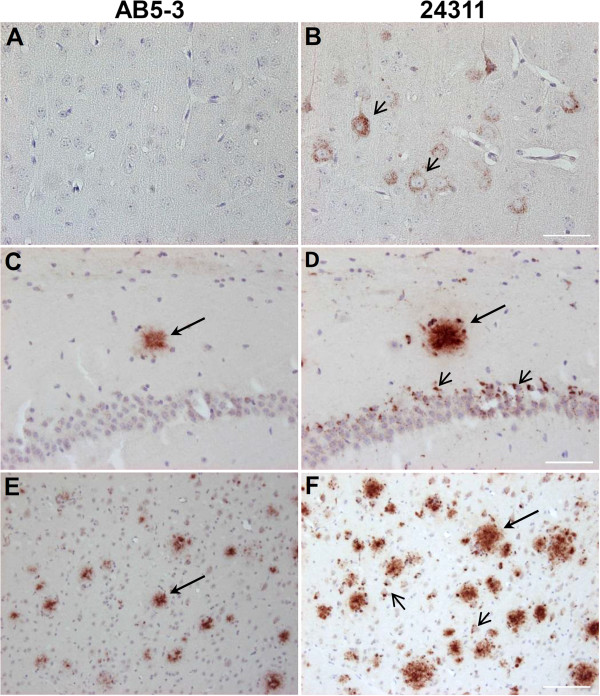
**Young 5XFAD and APP/PS1K1 mice do not present intraneuronal Aβ**_**5-x **_**deposits.** No intraneuronal Aβ_5-x_ deposits were found in cortical brain sections of 1.5-month-old 5XFAD homozygous mice **(A)**, as well as in hippocampal **(C)** and cortical sections of 2-month-old APP/PS1K1mice **(E)**. Parallel sections probed with the 24311 pan-Aβ antibody demonstrated the presence of intraneuronal Aβ immunoreactivity in both AD transgenic mouse models (small arrows: **B, D, F**). Abundant amyloid plaques were detected with both antibodies in young APP/PS1K1 mice (large arrows: **C-F**). Scale bar: A-D = 50 μm; **E, F** = 100 μm.

In order to find out whether or not intracellular Aβ_5-X_ is present early in the pathology, we used young APP/PS1KI (2-month-old) and homozygous 5XFAD (1.5-month-old) brain sections immunostained with the AB5-3 as both models harbor abundant intraneuronal Aβ in contrast to young 3xTg mice, which accumulate mostly APP [24,25]. No intracellular Aβ_5-X_ was evident, while the 24311 pan-Aβ antibody consistently showed intraneuronal aggregations in both mouse models. (Figure 7). Therefore, Aβ_5-X_ is not an early marker for neurodegeneration as it was only present in extracellular amyloid deposits.

Both diffuse and dense core plaques were positive. As it is known that neither 5XFAD, nor APP/PS1KI or 3xTg develops CAA, we could not find any blood vessels stained with the Aβ antibodies used.

## Discussion

Nowadays it is evident that a great heterogeneity of Aβ species exist that are deposited in AD brains including the full-length peptides, Aβ_1–40_ and Aβ_1–42_, and different C- and N- truncated isoforms (for example [26-28]). Especially N-truncated Aβ peptides experienced a renaissance in the last decade with the development of new transgenic mouse models and novel Aβ N-terminal specific antibodies for AD research [13,23]. N-truncated Aβ_x–42_ species have attracted much attention triggering the accumulation of Aβ into neurotoxic aggregates. Ancolio et al. [29] was the first to show a selective and drastic increase of these species triggered by the mutation V715M APP770 in the APP gene, postulating that all Aβ_x–42_ variants are main factors driving AD pathology.

The earliest evidence however for N-truncated peptides was observed by Masters et al. [30] demonstrating that Aβ peptides starting with phenylalanine at position 4 are main components of amyloid plaques. Aβ_4–42_ has been suggested to play an important role in the disease since it is deposited in early stages of the neuropathology before other isoforms appear [31]. On the other hand, another prominent N-truncated variant, Aβ_pE3–42_, was also suggested as a key player because of its limited degradation and remarkable stability [32]. It is becoming more and more accepted that the most prevalent Aβ variants present in the brain regions affected by the disease are mainly the 42 ending variants Aβ_1–42_, Aβ_pE3–42_ and Aβ_4–42_ (for example [26,28,33]). These Aβ variants, particularly Aβ_4–42_ and Aβ_pE3–42,_ are considered to be the most toxic due to their biochemical propensities to rapidly form stable oligomers [23].

Despite the above mentioned importance of N-truncated peptides for AD, little is known about Aβ_5–X_. It has been recognized to be present in Aβ deposits of SAD patients [34]. Using mass spectrometry and Western blot of SAD cases and mass spectrometry of SAD and FAD cases (M146V PS1 or KM670/671NL APP), Aβ_5–40/42_ was one of the detected N-truncated species [26-28,31]. Regarding transgenic mouse lines, mass spectrometry of immunoprecipitated Aβ peptides have also provided evidence of the presence of Aβ_5–42._ Casas et al. [20] reported Aβ_5–42_ deposition in the APP/PS1KI mice. Our group detected Aβ_5–42_ peptides in the 5XFAD mouse model [35].

The current report aims to provide a better understanding of a possible contribution of Aβ_5–X_ in three different transgenic amyloid mouse models, SAD and FAD cases. Previous papers reported on a rabbit polyclonal [34] and a mouse monoclonal antibody [14] specific to the N-terminal end of Aβ_5–40/42,_ which bind to the epitope Aβ_5–12_ (NH_2_-RHDSGYEVC-COOH). We have generated a rabbit polyclonal antibody, which specifically binds to Aβ_5–x_ as shown by dot blot and Western blot. The AB5-3 strongly detects Aβ_5–42_ low molecular weight oligomers showing its specificity for peptides starting with an arginine at position 5, while other Aβ variants starting at position 1, 3 (pyroglutamate) and 4 did not cross-react. While the polyclonal antibody AB5-3 reacted specifically with the free N-terminus of Aβ_5–42_, we cannot exclude that it also may recognize Aβ_6-X_ variants.

In agreement with the observations of the previously published polyclonal antibody [34], we detected parenchymal and vascular deposits in SAD. Vascular Aβ_5-x_ seems to be present in most (80%) of the cases. Confirming published observations [34], the vascular Aβ_5-x_ deposits often showed a stronger signal than aggregation in plaques of SAD cases. Immunostainings with the AB5-3 confirmed the presence of the Aβ_5–x_ in extracellular deposits, however at a minor degree corroborating previous studies.

For the first time we present evidence that Aβ_5-x_ immunoreactivity is abundant in extracellular plaques and vascular deposits in different FAD cases. The cases studied included subjects expressing the APP Arctic and Swedish mutations, as well as the PS1 delta exon 9 mutation. Different patterns of Aβ_5–X_ aggregation were found among the different FAD cases. Previous data obtained from patients carrying the APP Arctic mutation reported ring-like plaques with Aβ peptides showing an accentuation of the contour and negative or weakly stained centers [36]. According to Nilsberth et al. [16] the Arctic mutation leads to an increase of Aβ protofibril formation, however the underlying molecular mechanism is still unknown.

Intraneuronal Aβ is a major risk factor in AD pathology triggering neuron loss [37-40]. During the last years, intraneuronal accumulation has been reported in several mouse models including APP_SDL_PS1_M146L_[41], APP_SL_PS1_M146L_[42], Tg2576 [43], 3xTg-AD [19], APP_Arc_[44,45], 5XFAD [21], APP_T714I_[46], APP_SL_/PS1_M146L_[47], APP/PS1KI [20,48,49], TBA2 mice expressing pyroglutamate modified Aβ_3–42_[50] and in Tg4-42 expressing Aβ_4–42_[23]. Furthermore, early intraneuronal Aβ accumulations have been detected in the homozygous 5XFAD mouse model at 1.5 months of age immediately preceding extracellular plaque deposition occurring at the age of 2 months [51]. Abundant intraneuronal Aβ has been demonstrated to correlate with a reduced number of neurons irrespectively of the extracellular Aβ aggregates in APP/PS1KI and in 5XFAD mice [40].

Using the AB5-3 no Aβ_5-x_ intraneuronal immunoreactivity was found in young mice APP/PS1KI and in 5XFAD mice. This observation indicates that Aβ_5-x_ may not contribute to trigger neuronal degeneration and appears late in AD pathology. Despite the fact that Aβ_5–x_ is a scarce variant in AD brains it was present in almost all sporadic cases and in virtually all the familial AD cases tested with a different degree regarding CAA and plaque deposition. Contrasting previous findings with abundant intraneuronal N-truncated Aβ_4-x_ in homozygous 5XFAD mice [51], Aβ_5–x_ appeared late in the amyloid cascade as there was no intraneuronal staining in young APP/PS1KI and homozygous 5XFAD mice. A variety of different N-truncated Aβ peptides besides Aβ_5-x_ (Arg-5) have been identified in AD brains including Ala-2, pyroglutamylated Glu-3, Phe-4, His-6, Asp-7, Ser-8, Gly-9, Tyr-10 and pyroglutamylated Glu-11 [26-31,52-55]. We cannot rule out however that the polyclonal antibody AB5-3 may cross-react with other N-truncated Aβ variants not studied here that may be more prevalent than Arg-5 in amyloid plaques.

## Conclusions

Although a considerable amount of Aβ_5-x_ can be found in transgenic AD mouse models, SAD and FAD cases, no intracellular Aβ_5-x_ deposits of these peptides were found neither in the different mouse lines nor in the human cases. Due to the lack of intracellular Aβ_5-x,_ it is unlikely to participate in early events of the AD pathology. This indicates that Aβ_5-x_ might participate in extracellular precipitation of Aβ peptides within plaques and is therefore not likely to be involved in triggering neuronal loss.

## Competing interests

The authors declare that they have no competing interests.

## Authors’ contributions

EAG performed experiments and wrote the paper, YB, BCR and OW contributed to experiments, LL, MI, AP and AV-A contributed with neuropathological and clinical expertise, TAB designed the study and wrote the paper. All authors read and approved the final manuscript.
